# Public perception and attitude towards dengue prevention activity and response to dengue early warning in Malaysia

**DOI:** 10.1371/journal.pone.0212497

**Published:** 2019-02-28

**Authors:** Rafdzah Zaki, Siti Norsyuhada Roffeei, Yien Ling Hii, Abqariyah Yahya, Mahesh Appannan, Mas Ayu Said, Ng Chiu Wan, Nasrin Aghamohammadi, Noran Naqiah Hairi, Awang Bulgiba, Mikkel Quam, Joacim Rocklov

**Affiliations:** 1 Centre for Epidemiology and Evidence-Based Practice, Department of Social and Preventive Medicine, Faculty of Medicine, University of Malaya, Malaysia; 2 Epidemiology and Global Health, Department of Public Health and Clinical Medicine, Umea University, Umea, Sweden; 3 Centre for Occupational and Environmental Health, Department of Social and Preventive Medicine, Faculty of Medicine, University of Malaya, Malaysia; Universite des Montagnes, CAMEROON

## Abstract

An early warning system for dengue is meant to predict outbreaks and prevent dengue cases by aiding timely decision making and deployment of interventions. However, only a system which is accepted and utilised by the public would be sustainable in the long run. This study aimed to explore the perception and attitude of the Malaysian public towards a dengue early warning system. The sample consisted of 847 individuals who were 18 years and above and living/working in the Petaling District, an area adjacent to Kuala Lumpur, Malaysia. A questionnaire consisting of personal information and three sub-measures of; i) perception, ii) attitude towards dengue early warning and iii) response towards early warning; was distributed to participants. We found that most of the respondents know about dengue fever (97.1%) and its association with climate factors (90.6%). Most of them wanted to help reduce the number of dengue cases in their area (91.5%). A small percentage of the respondents admitted that they were not willing to be involved in public activities, and 64% of them admitted that they did not check dengue situations or hotspots around their area regularly. Despite the high awareness on the relationship between climate and dengue, about 45% of respondents do not know or are not sure how this can be used to predict dengue. Respondents would like to know more about how climate data can be used to predict a dengue outbreak (92.7%). Providing more information on how climate can influence dengue cases would increase public acceptability and improve response towards climate-based warning system. The most preferred way of communicating early warning was through the television (66.4%). This study shows that the public in Petaling District considers it necessary to have a dengue warning system to be necessary, but more education is required.

## Introduction

Dengue is transmitted by infected female mosquitoes; *Aedes albopictus* & *Aedes aegypti*, the latter being the primary vector. These mosquitoes feed in the daytime with the possibility of multiple-biting of people during the feeding period. A study in rural Thailand showed that 81% of multiple blood meals during the rainy season occurred among those living in the same house [1]. Symptoms of dengue fever usually lasts for 2–7 days, and often include high fever (up to 40°C/104°F), accompanied by one or several of the following manifestations: pain behind the eyes, severe headache, rash, muscle and joint pains, and vomiting [2].

Worldwide estimation of the dengue burden from 1990–2013 yields a range of 50–100 million dengue cases per year [3]. In Southeast Asia, dengue poses an immense disease and economic burden which exceeds that for upper respiratory infections and Japanese encephalitis [4]. In Malaysia, it is regarded as the most important communicable disease surpassing HIV/AIDS and tuberculosis [5]. In 2017, there were 83,374 dengue cases in Malaysia with more than half (n = 45,026, 54%) occurring in the state of Selangor [6].

Currently there is no specific treatment for dengue fever, but proper hospital care is essential in some cases [7]. Since the lack of effectiveness and the side-effects of the dengue vaccine makes vaccination a non-viable option for the moment, vector control and surveillance are still the dominant measures in dengue prevention [8].

Dengue transmission is influenced by various factors which include weather or climate, host immunity, vector capacity and dengue control effort [9]. Several studies have documented the link between climate and dengue transmission [10–13]. The influence of weather and climate variability on dengue fever is through their direct impacts on the biological or life cycles of *Aedes* mosquitoes and the length of the extrinsic incubation period of the dengue virus in mosquitoes [14]. The possibility of using weather predictors such as weekly mean temperature and cumulative rainfall to forecast weekly dengue incidence up to 16 weeks in advance has been demonstrated before [15]. Thus, a climate-based dengue early warning system could potentially be useful with ample time for the community to take necessary actions.

A prototype dengue early warning system was developed to produce probabilistic forecasts of dengue risk three months ahead of the 2014 World Cup in Brazil for over 550 ‘microregions’. The early warnings were disseminated to the general public and visitors travelling to Brazil [16]. Evaluation of this system suggested that an early warning model framework may be useful for public health services, not only ahead of mass gatherings, but also before the peak dengue season each year, to control potentially explosive dengue epidemics [16, 17],

Recently, a study in Singapore demonstrated the application of a forecasting tool in dengue control program [18]. This model comprises a “real-time” schedule, with data being updated weekly and predictions sent out to the Ministry of Health and the Environmental Public Health Operations Department of the National Environment Agency. Their forecasts have been accurate enough to guide public health interventions and hospital bed management. It has facilitated early risk communication to the public and the advanced launch of the annual Dengue Campaign two months ahead of its traditional launch [18]. Apart from that, the World Health Organization (WHO) has developed an early warning system algorithm and support system which is being tested in some countries. This further highlights the importance of our present study as the WHO system also needs to take into account factors of population’s acceptance, attitude and perceptions [19].

The main goal of a dengue early warning system would be to provide prior announcement to the public and government bodies of any possible dengue outbreak to allow proper preventive measures to be taken. For such a system to be effective, a timely and understandable communication of the warning and the capacity to act on the warning, particularly at the local level are crucial. As shown through other warning systems (e.g. famine, hurricanes), a warning system will not work if there is a lack of willingness and capacity to respond to it [20, 21]. A review of early warning systems conducted for World Vision has identified systemic barriers of converting information into actions [22]. Insufficient warning interpretation at the community level; a lack of guidelines for appropriate actions; disagreement on the accuracy and appropriateness of early warning systems; and a lack of understanding on coping strategies were among internal barriers identified in the review [22]. Therefore, the perception of the community towards dengue early warning and the willingness to take preventive measures in their home environment are important determinants of their participation in community-based programs in response to the early warning system. Thus, it is important to know the public’s perception, attitude and their response towards a dengue early warning system.

## Materials and methods

### Participants and study area

A cross-sectional study was conducted from May 2017 to July 2017 in the Petaling District which is situated in the Malaysian state of Selangor. Petaling District has one of the highest number of dengue cases in Malaysia. The target population were members of the public studying, working, or living in the Petaling District. Participants were selected from a few public places, such as shopping malls and post office, in order to capture participants from various backgrounds in the community. Participants were approached by enumerators at various times of the day during data collection. Only those who consented were included in this study. Those aged below 18 years old were excluded. A total of 1,000 dual language (English and Malay) self-administered questionnaires were distributed. Both local and foreigners were included in this study.

### Instrument

The questionnaire (S1 Text) was developed by this research team which consist of experts in public health, environmental health, and dengue modelling. The translation of the questionnaires from English to Malay language was done by two researchers from the University of Malaya and the final dual language questionnaire was reviewed by the entire research team. The team members checked the translated questionnaires and any differences raised were discussed and corrected accordingly. The final version of the questionnaire was pilot tested in a community with similarities to the study population.

The questionnaire consisted of a socio-demographic section and a three other sections. Section A) was on perception, Section B) on attitude and Section C) was on the response towards an early warning system. Section A (perception) consisted of 18 questions, where participants were asked about their knowledge of dengue infection, prevention and the effects of climate change on dengue. Section B (attitude) consisted of 14 questions on the attitude towards a dengue early warning system. Finally, Section C consisted of 18 questions on the response to and effectiveness of an early warning system.

The three sections consist of closed-ended questions with ‘yes’, ‘no’, ‘don’t know’, ‘not sure’, or a multiple choice question selection, where applicable. These sections have Cronbach’s alpha values of more than 0.60 (Section A = 0.716, Section B = 0.619, and Section C = 0.795). A review article on the application of Cronbach’s alpha has identified wide range of interpretation of alpha values. Most researchers described a value of >0.6 as acceptable and sufficient, with alpha coefficients that are less than 0.5 as unacceptable [23]. Thus the internal consistency of this questionnaire was considered acceptable. Test re-test reliability of the questionnaire was performed among a sample of 50 participants that had similar characteristics to the study population. The gap between the first and second testing was less than 5 days. Analysis using intraclass correlation coefficient (ICC) showed that all items achieved moderate to excellent reliability with ICC ranging from 0.6 to 1.0.

### Ethical approval

The study was approved by the Medical Ethics Committee of the University of Malaya Medical Centre (MECID: 20143–68). The respondents were given an explanation of the objectives and benefits of the study, with verbal and written consent obtained from those who agreed to participate.

### Data entry and analyses

All statistical analyses were performed using the IBM Corp. Released 2013. IBM SPSS Statistics for Windows, Version 22.0. Armonk, NY: IBM Corp. Double data entry was performed to ensure data quality. Data were entered into two separate files and then cross-checked for discrepancies. Any discrepancies and missing data were referred back to the original questionnaire (hard copy). Frequency and percentages was calculated for categorical variables (e.g. gender, race), while mean and standard deviation was calculated for continuous variables (e.g. age, income). The association between categorical variables were measured using chi-square test. P-values of < 0.05 were considered significant.

## Results

### Socio-demographic characteristics

Out of 1000 questionnaires being distributed, only 847 were filled completely, giving a response rate of 84.7%. Most of the respondents had never had dengue fever (80.8%) but do know people who had been infected with dengue (78.1%).

The respondents were mostly females (64.7%) with an average age of 26.90 ± 9.58 years old (age range: 18–71 years old). Almost all the participants were Malaysians (98.8%) and the majority were Malays (75.7%). Less than half of the respondents lived in the Petaling District (42.8%), while the rest of them were either studying or working in the district. Of those who lived in Petaling, only 35.8% had been living in the area for more than 5 years. Many participants had at least a university degree (46.9%) and most were single (70.0%). Most of the respondents were students (52.7%), the rest were private sector workers (19.7%), civil servants (13.9%), self-employed (9.6%), unemployed (3.3%) and others (0.8%). The majority of the respondents claimed never to have had dengue fever (80.8%), but have known persons who have been infected with dengue (78.1%). Two-thirds of the respondents had less than 5 people in their household (67.8%). Further details about the demographic data of the participants are shown in Table 1.

**Table 1:** Socio-demographic characteristics of participants (n = 847) in Petaling District as of July 2017.

Variable	Variable	N (%)
1. Gender (n = 846)	Male	299 (35.3)
	Female	547 (64.7)
2. Age (n = 823)	≤40 years old	751 (91.3)
	>40 years old	72 (8.7)
3. Nationality (n = 835)	Malaysian	825 (98.8)
	Non-Malaysian	10 (1.2)
4. Race (n = 826)	Malay	625 (75.7)
	Chinese	64 (7.7)
	Indian	94 (11.4)
	Others	43 (5.2)
5. Do you live in Petaling District? (n = 843)	Yes	361 (42.8)
	No	482 (57.2)
5(a). Do you study/work in Petaling District? (n = 635)	Yes	425 (66.9)
	No	210 (33.1)
5(b). Period living in Petaling District (n = 497)	< 1 year	110 (22.1)
	> 1–3 years	145 (29.2)
	> 3–5 years	64 (12.9)
	> 5 years	178 (35.8)
5(c). Do you own your current residence? (n = 524)	Yes	196 (37.4)
	No	328 (62.6)
6. What type of house do you currently reside in? (n = 834)	Individual house or bungalow	79 (9.5)
	Twin/semi-detached	58 (7.0)
	Terrace house	289 (34.7)
	Flat	112 (13.4)
	Apartment/condominium	185 (22.2)
	Shop house/long house/others	111 (13.2)
7. Highest education level (n = 838)	No formal education	3 (0.4)
	Primary school	5 (0.6)
	Secondary school	150 (17.9)
	Diploma	236 (28.2)
	Degree	393 (46.9)
	Master’s/PhD	51 (6.1)
8. Marital status (n = 842)	Single	589 (70.0)
	Married	239 (28.4)
	Divorced	9 (1.1)
	Widow/widower	5 (0.6)
9. Occupation (n = 837)	Student	441 (52.7)
	Self-employed	80 (9.6)
	Government workers	116 (13.9)
	Private workers	165 (19.7)
	Unemployed	28 (3.3)
	Other (retired)	7 (0.8)
10. Number of people in household (n = 818)	≤ 5 people > 5 people	555 (67.8) 263 (32.2)
11. Average monthly income (n = 458)	≤ MYR3000 > MYR3000	323 (70.5) 135 (29.5)
12. Average household monthly income (n = 562)	≤ MYR6000 > MYR6000	389 (69.2) 173 (30.8)

*MYR = Malaysian Ringgit

### Perception towards a climate-based dengue early warning

Table 2 summarizes the perception of the respondents towards an early warning system (Section A). The majority of respondents knew what dengue fever is (97.1%) and that it could lead to death (96.0%). More than half of them admitted to having sufficient knowledge on dengue prevention (64.1%) and would be very concerned on if they were to get an infection more than once (81.9%). Most agreed that mosquito repellents (68.2%) and removing breeding sites (83.7%) were important in protecting against dengue. The majority (90.6%) of respondents agreed that climate change affects human health and 68.9% agreed that global warming increases the chances of dengue outbreaks. They also agreed that the number of dengue cases increases after rainy days (76.2%), and that increasing temperature elevates the number of dengue cases in their area (57.8%). Finally, a majority of respondents thought that an early warning would be useful in informing the community so that timely preventive actions could be taken (80.6%).

**Table 2:** Perception towards an early warning system (Section A).

Variable		N (%)
1. Do you know what dengue fever is? (n = 841)	Yes	817 (97.1)
	No	24 (2.9)
2. Do you think dengue fever can cause mortality? (n = 844)	Yes	810 (96.0)
	No	11 (1.3)
	Don’t know	23 (2.7)
3. Do you think you and your family members could be infected with dengue fever? (n = 836)	Yes	661 (79.1)
	No	62 (7.4)
	Don’t know	113 (13.5)
4. In your opinion, what is your risk of being infected with dengue fever? (n = 838)	Low	188 (22.4)
	Medium	457 (54.5)
	High	193 (23.0)
5. Do you think you have sufficient knowledge of the ways to prevent yourself from dengue infection? (n = 844)	Yes	541 (64.1)
	No	208 (24.6)
	Don’t know	95 (11.3)
6. Do you think the dengue situation is serious in the area you live in? (n = 845)	Yes	384 (45.4)
	No	296 (35.0)
	Don’t know	165 (19.5)
7. Do you think it is possible to be infected with dengue many times? (n = 845)	Yes	418 (49.5)
	No	188 (22.2)
	Don’t know	239 (28.3)
8. How concerned would you be if it was the second time or more for your parents/children to be infected with dengue? (n = 843)	Very concerned	690 (81.9)
	Concerned	112 (13.3)
	Slightly concerned	31 (3.7)
	Not concerned	10 (1.2)
9. Which methods can be used to protect yourself and your family members from dengue infection? (You may tick several options) (n = 846)		
9.1) Nothing	Yes	8 (0.9)
	No	838 (99.1)
9.2) Don’t know	Yes	33 (3.9)
	No	813 (96.1)
9.3) Mosquito repellent	Yes	577 (68.2)
	No	269 (31.8)
9.4) Insecticide	Yes	352 (41.6)
	No	494 (58.4)
9.5) Bed nets	Yes	395 (46.7)
	No	451 (53.3)
9.6) Remove mosquito breeding sites	Yes	708 (83.7)
	No	138 (16.3)
9.7) Others	Yes	116 (13.7)
	No	730 (86.3)
10. Do you think the global climate is changing? (n = 845)	Yes	708 (83.8)
	No	43 (5.1)
	Don’t know	94 (11.1)
11. Do you think the climate change does not influence Malaysia climate? (n = 844)	Yes	189 (22.4)
	No	503 (59.6)
	Don’t know	152 (18.0)
12. Do you think the climate change affects human health? (n = 844)	Yes	765 (90.6)
	No	25 (3.0)
	Don’t know	54 (6.4)
13. Do you think the global warming could increase the risk of dengue outbreaks? (n = 842)	Yes	580 (68.9)
	No	73 (8.7)
	Don’t know	189 (22.4)
14. Do you think the climatic factors may affect the life cycle of mosquitoes but not dengue cases? (n = 841)	Yes	405 (48.2)
	No	198 (23.5)
	Don’t know	238 (28.3)
15. Do you think the number of dengue cases increases after rainy days? (n = 842)	Yes	642 (76.2)
	No	64 (7.6)
	Don’t know	136 (16.2)
16. Do you think the increasing temperature elevates the number of dengue cases in your area? (n = 843)	Yes	487 (57.8)
	No	110 (13.0)
	Don’t know	246 (29.2)
17. Do you think the information about previous temperature and rainfall can be used to predict dengue outbreak in future? (n = 842)	Yes	506 (60.1)
	No	87 (10.3)
	Don’t know	249 (29.6)
18. Do you think an early warning is a useful tool for community to take preventive actions to avoid possible infection within sufficient time? (n = 841)	Yes	678 (80.6)
	No	63 (7.5)
	Don’t know	100 (11.9)

### Attitudes towards a climate-based dengue early warning

The majority of respondents wanted to help reduce the number of dengue cases in their area (91.5%). They also agreed that an early warning system was important in preventing an outbreak (94.4%) and that an advanced warning system helps in avoiding potential dengue infections (90.5%). The community needs public education about the warning system (86.6%) and want to know more about how changes in climate can be used to predict a dengue outbreak (92.7%). Most respondents would like to receive a periodical update on information of dengue early warning (91.7%) and they chose the television (66.4%) as a method to receive early warning of dengue (Table 3).

**Table 3:** Attitude towards an early warning system (Section B).

Variable		N (%)
19. I want to help to reduce number of dengue cases in my area (n = 844)	Yes	772 (91.5)
	No	12 (1.4)
	Not sure	60 (7.1)
20. An early warning is important for the prevention of dengue outbreak (n = 843)	Yes	796 (94.4)
	No	19 (2.3)
	Not sure	28 (3.3)
21. It is possible to predict dengue outbreak using climate (n = 843)	Yes	461 (54.7)
	No	80 (9.5)
	Not sure	302 (35.8)
22. A warning of dengue in advance helps us to avoid potential dengue infections (n = 844)	Yes	764 (90.5)
	No	23 (2.7)
	Not sure	57 (6.8)
23. I will only believe a dengue early warning if it is based on risk factors other than climate (n = 840)	Yes	426 (50.7)
	No	414 (49.3)
24. I will believe an early warning only if the information is provided by the government agency (n = 843)	Yes	494 (58.6)
	No	349 (41.4)
25. The government agency should include information of early warning of dengue outbreak as and when they update dengue situations for the public (n = 838)	Yes	776 (92.6)
	No	16 (1.9)
	Not sure	46 (5.5)
26. We do not need an early warning since weekly dengue situations for my area is available online or social media (n = 839)	Yes	112 (13.3)
	No	556 (66.3)
	Not sure	171 (20.4)
27. It is a waste of time and efforts on dengue control if the predicted risk of dengue outbreak does not come true (n = 839)	Yes	129 (15.4)
	No	571 (68.1)
	Not sure	139 (16.6)
28. It is pointless for me to take action even with early dengue warning since my neighbours will not (n = 836)	Yes	232 (27.8)
	No	604 (72.2)
29. The community in my area needs public education about dengue early warning (n = 835)	Yes	723 (86.6)
	No	41 (4.9)
	Not sure	71 (8.5)
30. I want to know more about how climate can be used to predict a dengue outbreak (n = 834)	Yes	773 (92.7)
	No	61 (7.3)
31. I would like to receive a periodical update on information of dengue early warning (n = 833)	Yes	764 (91.7)
	No	69 (8.3)
32. In what way, would you like to receive an early warning for dengue? (n = 836)		
32.1) Mobile app	Yes	354 (42.3)
32.2) SMS	Yes	301 (36.0)
32.3) Radio	Yes	407 (48.7)
32.4) Television	Yes	555 (66.4)
32.5) Facebook	Yes	414 (49.5)
32.6) Twitter	Yes	239 (28.6)
32.7) Instagram	Yes	261 (31.2)
32.8) Other media	Yes	71 (8.5)
Email		8 (19.0)
Newspaper		18 (42.9)
Official announcements/news/campaigns/pamphlets		9 (21.4)
Social messaging apps (e.g. We Chat, WhatsApp)		7 (16.7)

### Response towards a climate-based dengue early warning

Table 4 shows the response of the respondents towards an early dengue warning system. Most respondents do not check the dengue situation in their area (64.6%) but are ready to take extra action if the dengue risk in their area increases (87.5%). After receiving an early warning of dengue outbreak, most will avoid outdoor activities at dawn/dusk (83.6%) and will share the information with others (92.5%).

**Table 4:** Response towards an early warning system (Section C).

Variable		N (%)
33. I check current dengue situations or hotspots around my area regularly (n = 838)	Yes	297 (35.4)
	No	541 (64.6)
34. I do not know what to do if someone informs me that it is very likely to have a dengue outbreak in the near future (n = 837)	Yes	241 (28.8)
	No	366 (43.7)
	Not sure	230 (27.5)
35. I will take extra action to prevent dengue infection if I know the risk of dengue is increasing in my area (n = 838)	Yes	733 (87.5)
	No	32 (3.8)
	Not sure	73 (8.7)
36. After I receive an early warning of dengue outbreak from the government agency, I will		
36.a) Increase source reduction activities (n = 832)	Yes	590 (70.9)
	No	77 (9.3)
	Not sure	165 (19.8)
36.b) Avoid outdoor activities at dawn or dusk (n = 836)	Yes	699 (83.6)
	No	70 (8.4)
	Not sure	67 (8.0)
36.c) Share information with others (n = 837)	Yes	774 (92.5)
	No	25 (3.0)
	Not sure	38 (4.5)
36.d) Request chemical fogging (n = 836)	Yes	664 (79.4)
	No	58 (6.9)
	Not sure	114 (13.6)
36.e) Call local authorities (n = 835)	Yes	542 (64.9)
	No	125 (15.0)
	Not sure	168 (20.1)
36.f) Use mosquito net (n = 837)	Yes	538 (64.3)
	No	167 (20.0)
	Not sure	132 (15.8)
37. I need to know how severe the predicted dengue outbreak will be in order to decide whether preventive measures are required (n = 840)	Yes	606 (72.1)
	No	145 (17.3)
	Not sure	89 (10.6)
38. I will stop action to prevent dengue infection if I know the risk of dengue in my area is low (n = 840)	Yes	211 (25.1)
	No	629 (74.9)
39. The government agency will conduct mosquito control program after they receive an early warning of dengue, so individual household does not need to do anything (n = 837)	Yes	193 (23.1)
	No	524 (62.6)
	Not sure	120 (14.3)
40. Removal of mosquito breeding sites at my premises will reduce the chance of dengue infections among my family members (n = 837)	Yes	679 (81.1)
	No	82 (9.8)
	Not sure	76 (9.1)
41. The local authority has already provided sufficient effort on dengue control in my area (n = 839)	Yes	368 (43.9)
	No	249 (29.7)
	Not sure	222 (26.5)
42. Chemical fogging by the local authority is good enough for us to prevent from dengue infection (n = 837)	Yes	316 (37.8)
	No	347 (41.5)
	Not sure	174 (20.8)
43. It is not my responsibility to remove mosquito breeding sites in my residence (n = 838)	Yes	135 (16.1)
	No	703 (83.9)
44. It is the responsibility of my family member to remove mosquito breeding sites in my residence (n = 839)	Yes	732 (87.2)
	No	107 (12.8)
45. It is necessary to continue the removal of mosquito breeding at home even during the period when there’s no dengue outbreak (n = 838)	Yes	766 (91.4)
	No	24 (2.9)
	Not sure	48 (5.7)
46. I can help to reduce dengue cases in my area by removing mosquito breeding sites at home (n = 839)	Yes	763 (90.9)
	No	29 (3.5)
	Not sure	47 (5.6)
47. Dengue outbreak in my community can be controlled if every household is committed to remove mosquito breeding sites (n = 839)	Yes	780 (93.0)
	No	17 (2.0)
	Not sure	42 (5.0)
48. I will take part in a public activity for dengue control or removal of mosquitoes breeding sites (n = 836)	Yes	672 (80.4)
	No	35 (4.2)
	Not sure	129 (15.4)
49. In my opinion, who should be responsible for preventing the spread of dengue disease? (You may tick several options) (n = 837)		
49.1) Health authority	Yes	707 (84.5)
49.2) Local council	Yes	595 (71.1)
49.3) Community leaders	Yes	551 (65.8)
49.4) Every household	Yes	707 (84.5)
49.5) I don’t think control actions are needed	Yes	27 (3.2)
50. In your opinion, what is the most effective method to reduce dengue infections in your area? (n = 838)		
50.1) Search and destroy mosquito breeding sites	Yes	726 (86.6)
50.2) Prevent from mosquito bites	Yes	260 (31.0)
50.3) Chemical fogging	Yes	440 (52.5)
50.4) Don’t know	Yes	26 (3.1)

The majority of respondents agreed that removal of mosquito breeding sites in their premises would reduce the chance of dengue infections among their family members (81.1%) and that it was their responsibility to remove mosquito breeding sites in their residence (87.2%). They mostly agreed that outbreaks could be controlled if every household played a role in removing breeding sites (93.0%). They were also willing to participate in a public activity for dengue control or removal of mosquitoes breeding sites (80.4%). The respondents thought that both the health authority (84.5%) and the household (84.5%) should be responsible for preventing the spread of dengue disease, through the search and destruction of mosquito breeding sites (86.6%).

### Socio-demographic factors associated with (i) Perception of the usefulness of an early warning for community to take timely preventive action; and (ii) Knowledge on actions to be taken following a notice on future dengue outbreak

**Fig 1:**
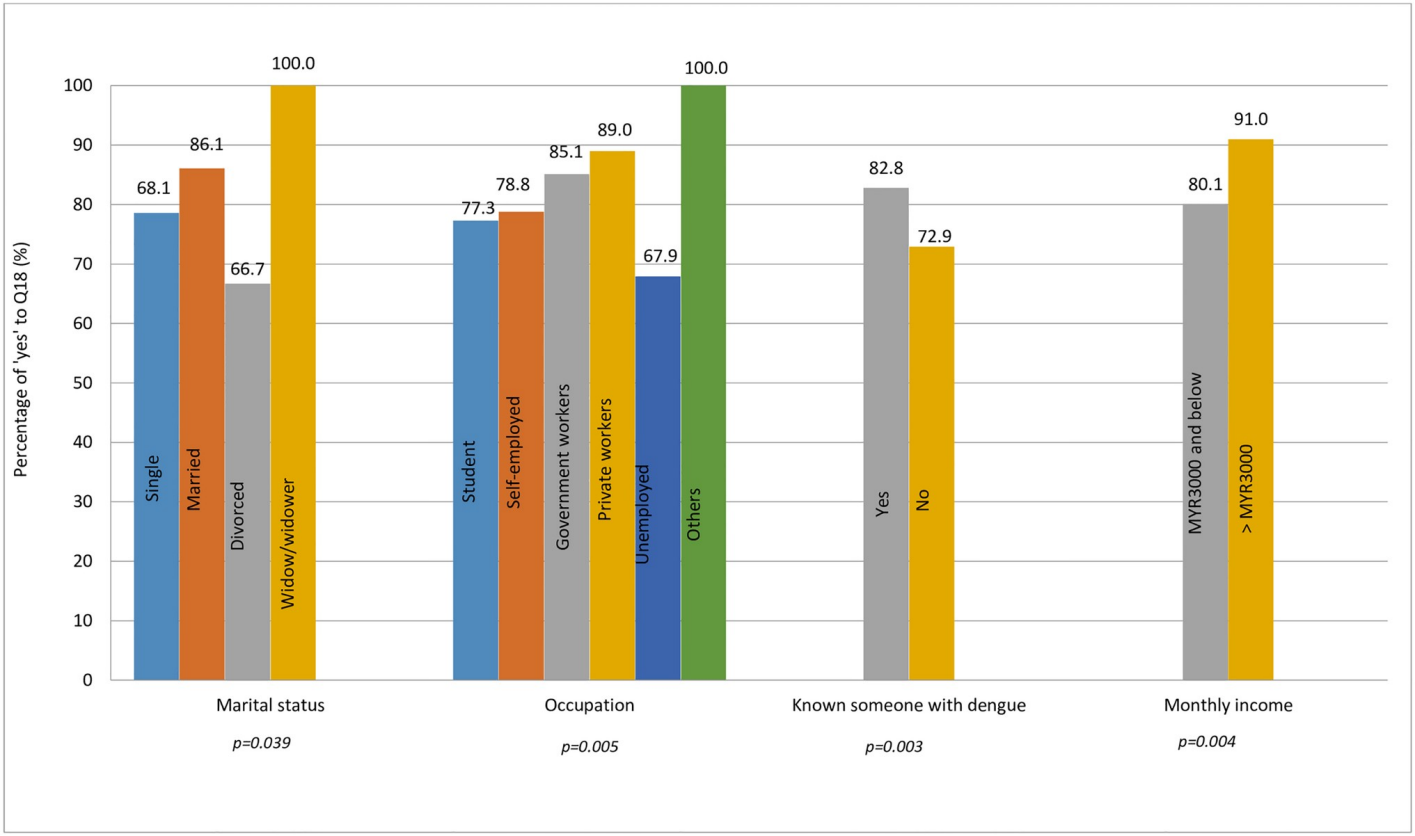
Significant demographic factors associated with the usefulness of an early warning to take timely preventive action.

Of the demographic variables, there is a significant association between marital status, occupation, knowing someone who’s been infected with dengue, and average monthly income with Q18 (the perception that an early warning is a useful tool for community to take preventive actions to avoid possible infection within sufficient time) (Fig 1).

Living in the Petaling District was the only significant factor associated with Q34 (the respondents’ knowledge on what to do if there is information regarding future dengue outbreak) with p-value of 0.004.

### Association of Section A with (i) Perception of the usefulness of an early warning for community to take timely preventive action; and (ii) Knowledge on actions to be taken following a notice on future dengue outbreak

**Fig 2:**
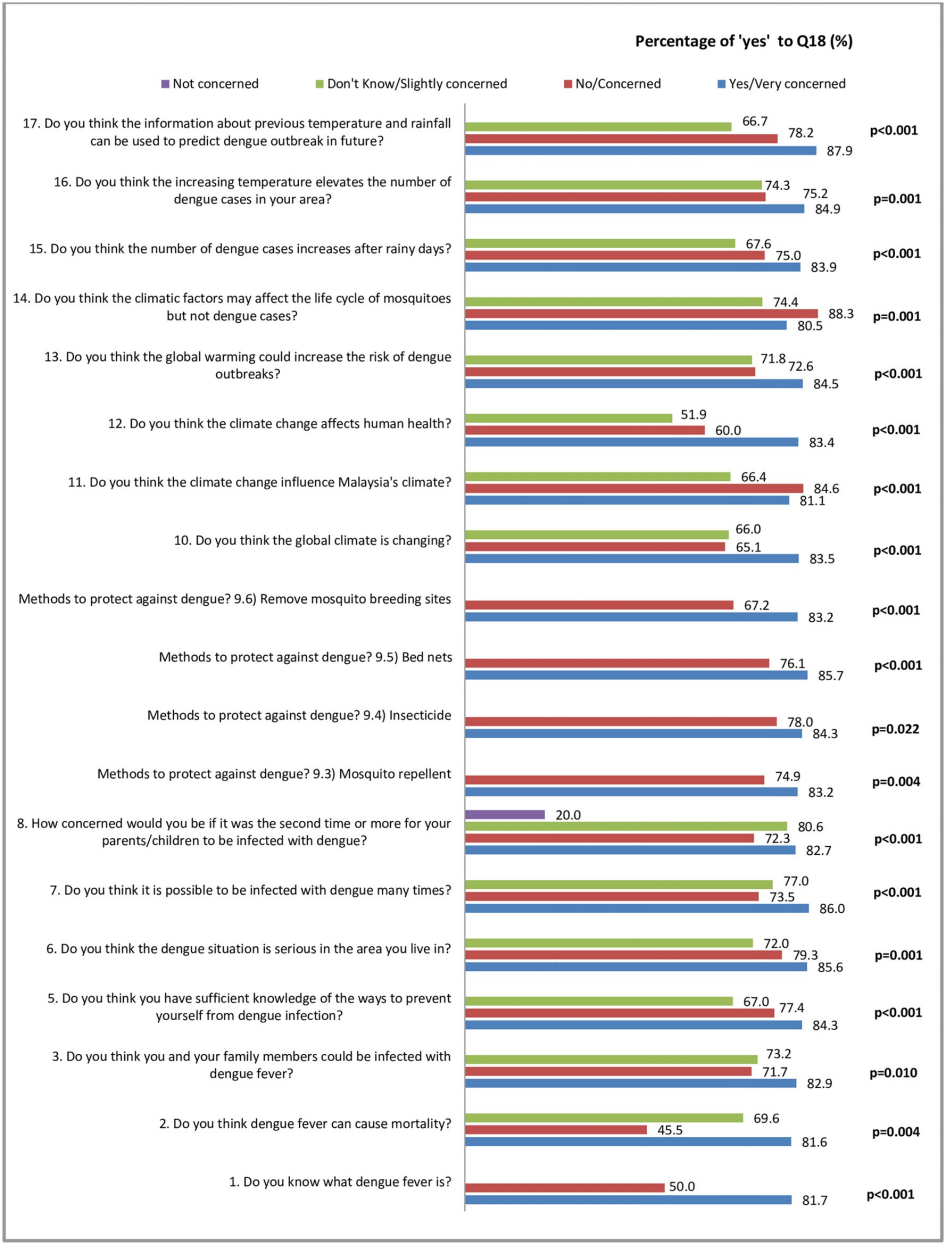
Significant factors in Section A associated with the usefulness of an early warning to take timely preventive action.

Most of the questions under perception (Section A) showed significant associations with Q18 (the perception that an early warning is a useful tool for community to take preventive actions to avoid possible infection within sufficient time) (Fig 2).

**Fig 3:**
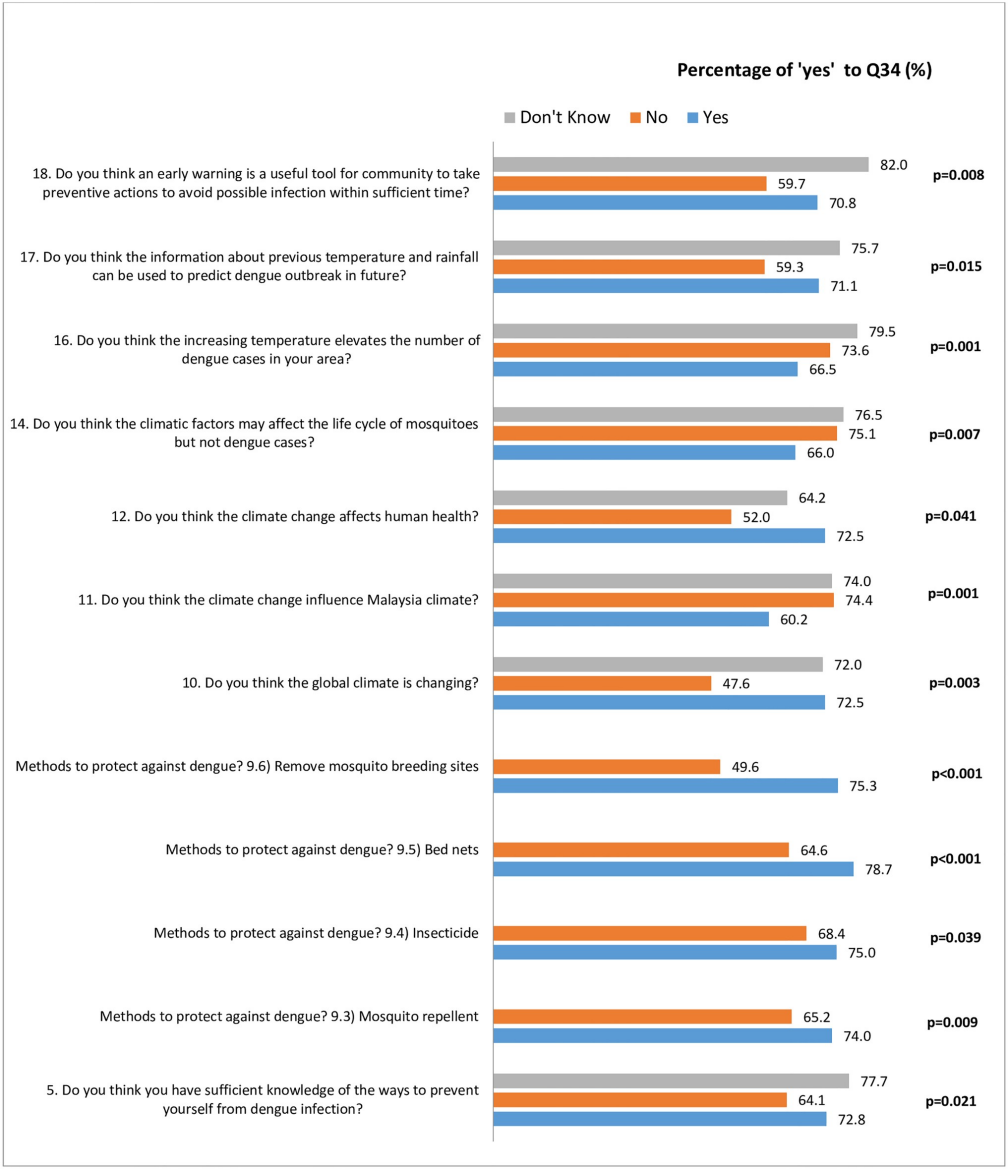
Significant factors in Section A associated with knowledge on actions to be taken following a notice on future dengue outbreak.

A similar pattern of association can also be seen with Q34 (the respondents’ knowledge on what to do if there is information regarding future dengue outbreak) (Fig 3).

## Discussion

### Knowledge on dengue and early warning system

In this study, more than half (64.1%) of the respondents think that they have sufficient knowledge to prevent dengue. They chose removal of breeding sites and mosquito repellent as the most effective methods in preventing dengue. Students and those living in the rural areas of Malaysia also have previously been shown to have good knowledge about dengue [24,25]. In terms of attitude, we found that the respondents wanted to help in reducing dengue cases. They will also share dengue information with others and will avoid outdoor activities at dawn/dusk. However, there was a small percentage of the respondents who admitted to not willing to be involved with public activities. This study also found that 64% of the respondents revealed that they did not check dengue situations or hotspots around their area regularly. This is important as public ignorance have been associated with the spread of dengue epidemics [26]. A study in China showed that participation of both the community and related department have made an exceptional difference in their dengue outbreak control. This participation includes leadership enhancement (by having a dengue control committee), daily risk assessment, and the elimination of mosquito/larvae breeding sites in high risk areas [27].

Most respondents agreed that early warning was important for the prevention of dengue outbreak; however more education about the warning system was needed. We also found that the respondents chose television as a way to receive dengue early warning. This is consistent with previous studies in Malaysia where the respondents chose television/radio as their main information source about dengue [25,28]. A good and continuous campaign about dengue prevention practices among the public is necessary as there is no specific treatment for dengue. Efforts taken by the government would be useless if there is no action taken by the public themselves. This highlights the importance of preventive actions against dengue to be made by both the public and the health authorities in Malaysia. Having better knowledge of dengue was associated with better dengue prevention practices among Malaysian public [29], including the Orang Asli (aboriginal) community [30].

### Dengue and climate

Results revealed that the respondents knew that climatic changes affect human health and that the global warming increases the chances of dengue outbreaks. They were aware that rainy days and increasing temperature contributed to an increase in dengue cases. Association between climatic factors (rainfall, humidity, temperature) and increasing dengue cases have been reported in previous studies [9, 31–34]. The acceleration of mosquito development stages following an elevation in temperature increases dengue transmission. Furthermore the pooling of water due to increased rainfall contributes to an increase in the number of breeding sites for mosquitoes [15]. Humid weather allows results in perfect conditions for dengue vectors to flourish [35]. A study in Thailand showed that humidity amplified the viable transmission range of dengue at a certain range of temperature—80% of dengue cases occurred at mean temperatures of 27–29.5°C and mean humidity of >75% [36]. A study by Cheong *et al* (2013) in Malaysia reported a positive association between the relative risk of dengue cases with, i) increased minimum temperature of 25.4–26.5°C with delay in effect by 51 days, and ii) bi-weekly accumulated rainfall of 215–302 mm with delay in effect by 26–28 days [37].

Despite having awareness on relationship between climate and dengue, about 45% of respondents do not know or are not sure this can be used to predict dengue. Providing more information on how climate can influence dengue cases would increase public acceptability and improve response towards a climate-based warning system.

### Dengue early warning system

Generally, the objective of a disease warning system is to prepare both the public health officials and the community with as much prior information as possible about the probability of a dengue outbreak, highlighting particular areas where it is likely to take place, so that the best response action can be planned [38]. By having a prior alert, it is hoped that policy makers would be able to draft the best preventive/management plan to face an upcoming predicted outbreak and reduce unnecessary fogging in some areas.

Through this study, we found that the respondents agreed that an advanced warning from the system would be useful in avoiding dengue infections by allowing them more time to take preventive actions (e.g. removing breeding sites). They are ready to take action should there be an increased dengue risk in their community.

Due to dengue’s high health and economic burden to the country, having an early warning system would allow for more cost-effective and efficient vector control efforts [19]. In Singapore, 42–59% of its total dengue economic burden cost are dedicated for control measures [39]. It was reported that in 2010, Malaysia spent a total of US$73.5 million (0.03% of Malaysia’s GDP) on its National Dengue Vector Control Program. About 92% of the amount was used for fogging [40].

Having early notice of a dengue outbreak will permit a timely action/response among the health personnel and the public, ahead of the predicted outbreak period. Alternatively, using freely available climate data would also contribute to reducing the cost of developing a warning system for developing countries. The system would also be an economical approach in planning resources and prioritising high risk areas for intensive preventive activities [9, 39].

Colombia and Singapore have published findings of their early warning systems. Colombia’s dengue early warning system managed to detect 75% of the total dengue outbreaks 1–5 months in advance, and missed some 12.5% cases. The system also classified western Colombia as a high risk area due to its dense population and the suitability of the climate conditions for the mosquitoes [41]. Meanwhile, in Singapore, its warning system forecasted an outbreak with a 3-month lag through the LASSO-derived models in their 2013 dengue control program, allowing advanced outbreak response preparations [18].

In this study, we also looked at the respondent’s perception and attitude towards the warning system. Most respondents did not check the dengue situation in their area, but they were ready to take extra actions if the dengue risk in their area increases. This suggests that a warning system on dengue risk could trigger the respondents to take preventive actions against dengue. It is helpful to know that there is a demand for such a system among the general public, as can be seen in this study. The demand would mean that the authority will have to provide such a system as a preventive measure. It is hoped that the public will respond appropriately towards an alert, so that efforts/work put into creating a system would not go to waste. Apart from that, having a demand would hopefully make it easier to get cooperation from the community and the government involving matters of campaign and other issues regarding the dengue warning system.

Although information does not automatically lead to positive decision making, it is hoped that the more information the public has about an early warning system, the more ready they will be to respond to it. An early warning system that is focused solely on information collection has been proven to not actually bring about early action [22]. Innovative approaches need to be explored by engaging multiple stakeholders to improve public response. In addition, involving the community in risk analysis, action planning and feedback on successes and challenges of early warning systems could reduce barriers to translating early warning into early action [22].

### Factors associated with Q18 (Perception of the usefulness of an early warning for community to take timely preventive action) and Q34 (Knowledge on actions to be taken following a notice on future dengue outbreak)

Our findings showed that respondents who were divorced, unemployed and earning below MYR3000 did not think that an early dengue warning would be useful for the community to take timely preventive action. This could be due to a lower education status and consequently lower dengue knowledge. Those who have known people who have been infected with dengue thought that an early dengue warning would be useful for the community to take timely preventive action.

Our data revealed that people who do not live in the Petaling District have the knowledge on what to do if they were informed of a possible future dengue outbreak. This could be partly due to the fact that most respondents (60.7%) who do not live in Petaling District were students; with half of them being degree holders. They thus have better knowledge about dengue preventive action.

We also found that having knowledge about dengue, having knowledge on dengue preventive actions and knowing about dengue and climate, too, were associated with the thought that an early dengue warning would be useful for the community to take timely preventive action. This is important as active community involvement is imperative in ensuring response towards dengue warning and successful dengue prevention. Active mobilisation of the community can only be effective and sustainable with adequate knowledge and support from the authority. Continuous education and monitoring by relevant organizations should be done to ensure long-term behavioural changes among the society towards dengue prevention [42].

Subsequently, those who have sufficient knowledge on dengue prevention, and those who have knowledge about dengue and climate would know what to do following information of a possible dengue outbreak. Our results thus supports the outcome reported by Chandren *et al* (2015) which stated that health and educational programs should focus on enhancing dengue knowledge as to increase dengue prevention practices [31]. A study in Costa Rica reported the association of positive breeding sites with lesser knowledge of dengue symptoms, lower education level, and lower ratings in (i) the importance of preventive actions and (ii) dangers of dengue [42].

### Strength and limitation

This is the first study looking at the public perceptions and attitudes towards a climate-based dengue early warning in dengue endemic country. Information from this study will be useful in exploring community needs before establishing early warning system for dengue. However, selection of study areas and participants in this study were based on non-probabilistic sampling. This might have caused some bias in the representation of our respondents. Causal relationship also cannot be established due to the cross-sectional nature of this study.

In this study, more than half of the respondents were females (64.7%) and Malays (75.7%). This is different from the population distribution in Petaling district according to the latest population census with 51.2% female and 52.6% Malay [43]. However the census was conducted in 2010, and data from the census only includes those residing in the district in contrast to the sample this study.

## Conclusion

In conclusion, members of the public in the Petaling District were aware of dengue and have some knowledge of dengue. They were also aware of the relationship between climate change and dengue incidence, and would like to receive more information about how climate data can be used to develop an early dengue warning system. Our findings indicated that the public in Petaling District do think it is necessary to have an early warning system. We recommend that the implementation of a dengue warning system will need to be accompanied by public educational programs for the warning system to work effectively. Having an early dengue warning system would go some way to reduce the high economic and health burden of dengue in Malaysia.

## Supporting information

S1 Text Questionnaire. (PDF)

S1 Table Association of socio-demographic factors with Q18 and Q34. (DOCX)

S2 Table Association of perception (Section A) with Q18 and Q34. (DOCX)
