# Supplementary material for: Public perception and attitude towards dengue prevention activity and response to dengue early warning in Malaysia
Source: PLoS One. 2019 Feb 28;14(2):e0212497. doi: 10.1371/journal.pone.0212497 (PMC6394956; doi:10.1371/journal.pone.0212497)
Supplement: S1 Text — (PDF) [file pone.0212497.s001.pdf]

Sample ID **Form #**

PERSONAL INFORMATION *MAKLUMAT PERIBADI*

***(Only tick one option on the questions unless other is stated** Sila pilih satu jawapan sahaja, kecuali jika dinyatakan lain)*

1. Gender *Jantina*<br>☐Female *Perempuan*<br>☐Male *Lelaki*	2. Age *Umur*:
3. Nationality *Warganegara*<br>☐Malaysian *Malaysia*<br>☐Non Malaysian *Bukan Malaysia*	4. Race *Bangsa*<br>☐Malay *Melayu* ☐Chinese *Cina*<br>☐Indian *India* ☐Others *Lain-lain*
5. Do you live in Petaling district? *Adakah anda menetap di daerah Petaling?*<br>☐Yes *Ya*<br>☐No *Tidak* (Go to Q6, *pergi ke Q6*)	5(a). Do you study/work in Petaling district? *Adakah anda belajar/bekerja di daerah Petaling?*<br>☐ Yes *Ya* ☐ No *Tidak*<br><br>5(b). Period living in Petaling district *Jangkamasa telah menetap di daerah Petaling*<br>☐ < 1 yr *< 1 tahun* ☐ ≥ 1-3 yrs *≥ 1-3 tahun*<br>☐ > 3-5 yrs *> 3-5 tahun* ☐ > 5 yrs *> 5 tahun*<br><br>5(c). Do you own current residence? *Adakah anda memiliki rumah yang anda diami?*<br>☐ Yes *Ya* ☐ No *Tidak*
6. What type of house you currently reside? *Apakah jenis rumah yang anda diami sekarang?*<br>☐Individual house or bungalow *Rumah sebuah atau banglo*<br>☐Twin/semi-detached house *Rumah berkembar*<br>☐Terrace house *Rumah teres atau berangkai*<br>☐Flat *Rumah pangsa*<br>☐Apartment/condominium *Apartmen/Kondominium*<br>☐Shophouse *Rumah kedai*<br>☐Long house *Rumah panjang*<br>☐Others *Lain-lain*	7. Highest education level *Tahap pendidikan tertinggi*<br>☐No formal education *Tiada pendidikan formal*<br>☐Primary school *Sekolah rendah*<br>☐Secondary school *Sekolah menengah*<br>☐Diploma *Diploma*<br>☐Degree *Ijazah*<br>☐Master/PhD *Master/PhD*

8. Marital status<br>*Taraf perkahwinan*<br>☐Single *Bujang*<br>☐Married *Berkahwin*<br>☐Divorced *Bercerai*<br>☐Widow/Widower *Duda/janda*	9. Occupation *Pekerjaan*<br>☐Student *Pelajar*<br>☐Self-employed *Bekerja sendiri*<br>☐Government workers *Pekerja kerajaan*<br>☐Private workers *Pekerja swasta*<br>☐Unemployed *Tidak berkerja*<br>☐Other *Lain-lain*___________________
10. Have you had dengue fever before?<br>*Pernahkah anda mendapat demam denggi*?<br>☐ Yes *Ya* ☐ No *Tidak*	11. Do you know any persons who have been infected with dengue?<br>*Adakah anda kenal sesiapa yang pernah dijangkiti denggi*?<br>☐ Yes *Ya* ☐ No *Tidak*
12. Number of people in your household<br>*Jumlah orang yang menetap di kediaman anda*<br>______________________________	12(a) State age of each individual live in your household<br>*Sila nyatakan umur setiap penghuni rumah anda*<br>______________________________
13. What is your average monthly income?<br>*Apakah purata pendapatan bulanan anda?*<br>______________________________	14. What is your average monthly household income?<br>*Apakah purata pendapatan isi rumah bulanan anda?*<br>______________________________

Terminology *Istilah*:

I. Dengue early warning: A warning of increasing dengue cases in the near future
Amaran awal denggi: Amaran tentang peningkatan kes denggi dalam masa terdekat

II. Climate-based early warning: A prediction of increasing dengue cases based on changes of temperature or rainfall patterns
Amaran awal berdasarkan iklim: Ramalan peningkatan kes denggi berdasarkan perubahan suhu atau corak hujan

A) PERCEPTION *PERSEPSI*:

1. Do you know what is dengue fever?
Adakah anda tahu apa itu demam denggi
☐Yes *Ya* ☐No *Tidak*

2. Do you think dengue fever can cause mortality?
Adakah anda rasa demam denggi boleh menyebabkan kematian?
☐Yes *Ya* ☐No *Tidak* ☐Don't know *Tidak tahu*

3. Do you think you and your family members could be infected with dengue fever?
Adakah anda rasa anda dan keluarga anda boleh dijangkiti demam denggi?
☐Yes *Ya* ☐No *Tidak* ☐Don't know *Tidak tahu*

4. In your opinion, what is your risk of being infected with dengue fever?
Pada pendapat anda, apakah risiko anda untuk dijangkiti demam denggi?
☐Low *Rendah* ☐Medium *Sederhana* ☐High *Tinggi*

5. Do you think you have sufficient knowledge of the ways to prevent yourself from dengue infection?
Adakah anda fikir anda mempunyai pengetahuan yang mencukupi tentang cara-cara untuk mengelakkan diri daripada jangkitan denggi?
☐Yes *Ya* ☐No *Tidak* ☐Don't know *Tidak tahu*

6. Do you think is the dengue situation serious in the area you live?
Adakah anda fikir situasi denggi serius di kawasan anda tinggal?
☐Yes *Ya* ☐No *Tidak* ☐Don't know *Tidak tahu*

7. Do you think is it possible you will be infected for dengue many times?
Adakah anda fikir anda boleh dijangkiti denggi dengan banyak kali?
☐Yes *Ya* ☐No *Tidak* ☐Don't know *Tidak tahu*

8. How concerned would you be if it was the <u>second time</u> or more for your parents/children to be infected with dengue?
Berapa bimbangkah anda jika ibubapa/anak-anak anda dijangkiti denggi untuk <u>kali kedua</u> atau lebih?
☐ Very concerned *Sangat bimbang* ☐ Concerned *Bimbang*
☐ Slightly concerned Sedikit bimbang ☐ Not concerned *Tidak bimbang*

9. Which methods can be used to protect yourself and your family members from dengue infection? (You may tick several options)
Kaedah-kaedah yang manakah boleh digunakan untuk melindungi diri anda dan ahli keluarga anda daripada jangkitan denggi? (Anda boleh menanda lebih daripada satu pilihan)
☐Nothing *Tiada* ☐Don't know *Tidak tahu* ☐Mosquito repellent *Penghalau nyamuk* ☐Insecticide *Racun serangga*
☐Bed nets *Kelambu* ☐Remove mosquitoes breeding sites *Penghapusan tempat pembiakan nyamuk* ☐Others *Lain-lain*

10. Do you think the global climate is changing?
Adakah anda rasa iklim global sedang berubah?
☐Yes *Ya* ☐No *Tidak* ☐Don't know *Tidak tahu*

11. Do you think the climate change does not influence Malaysia climate?
Adakah anda rasa perubahan iklim global tidak mempengaruhi iklim di Malaysia?
☐Yes *Ya* ☐No *Tidak* ☐Don't know *Tidak tahu*

12. Do you think the climate change affects human health?
Adakah anda rasa perubahan iklim mempengaruhi kesihatan manusia?
☐Yes *Ya* ☐No *Tidak* ☐Don't know *Tidak tahu*

13. Do you think the global warming could increase the risk of dengue outbreaks?
Adakah anda rasa pemanasan global boleh meningkatkan risiko untuk wabak denggi berlaku?
☐Yes *Ya* ☐No *Tidak* ☐Don't know *Tidak tahu*

14. Do you think the climatic factors may affect the life cycle of mosquitoes but not dengue cases?
Adakah anda rasa faktor iklim boleh mempengaruhi kitar hidup nyamuk tetapi bukan kes denggi?
☐Yes *Ya* ☐No *Tidak* ☐Don't know *Tidak tahu*

15. Do you think the number of dengue cases increases after rainy days?
Adakah anda rasa bilangan kes denggi akan meningkat selepas hujan beberapa hari?
☐Yes *Ya* ☐No *Tidak* ☐Don't know *Tidak tahu*

16. Do you think the increasing temperature elevates the number of dengue cases in your area?
Adakah anda rasa peningkatan suhu akan menambahkan bilangan kes denggi di kawasan anda?
☐Yes *Ya* ☐No *Tidak* ☐Don't know *Tidak tahu*

17. Do you think the information about previous temperature and rainfall can be used to predict dengue outbreak in future?
    Adakah anda rasa maklumat mengenai suhu dan hujan boleh digunakan untuk membuat ramalan wabak denggi di masa hadapan?
    ☐Yes *Ya* ☐No *Tidak* ☐Don't know *Tidak tahu*

18. Do you think an early warning is a useful tool for community to take preventive actions to avoid possible infection within sufficient time?
    Adakah anda fikir amaran awal adalah alat yang berguna bagi masyarakat untuk mengambil tindakan pencegahan bagi mengelakkan kemungkinan jangkitan denggi dalam tempoh masa yang mencukupi?
    ☐Yes *Ya* ☐No *Tidak* ☐Don't know *Tidak tahu*

B) ATTITUDE OF DENGUE EARLY WARNING *SIKAP UNTUK AMARAN AWAL DENGGI*

19. I want to help to reduce number of dengue cases in my area
    Saya mahu membantu mengurangkan bilangan kes denggi di kawasan saya
    ☐Yes *Ya* ☐No *Tidak* ☐Not sure *Tidak pasti*

20. An early warning is important for the prevention of dengue outbreak
    Amaran awal adalah penting untuk mencegah wabak denggi
    ☐Yes *Ya* ☐No *Tidak* ☐ Not sure *Tidak pasti*

21. It is possible to predict dengue outbreak using climate
    Wabak denggi boleh diramalkan dengan menggunakan iklim
    ☐Yes *Ya* ☐No *Tidak* ☐ Not sure *Tidak pasti*

22. A warning of dengue in advance helps us to avoid potential dengue infections
    Amaran awal denggi dapat membantu untuk mengelakkan potensi mendapat jangkitan denggi
    ☐Yes *Ya* ☐No *Tidak* ☐ Not sure *Tidak pasti*

23. I will only believe a dengue early warning if it is based on risk factors other than climate
    Saya hanya akan mempercayai amaran awal denggi sekiranya ramalan dibuat berdasarkan faktor-faktor selain daripada iklim
    ☐Yes *Ya* ☐No *Tidak*

24. I will believe an early warning ONLY IF THE INFORMATION is provided by the government agency
    Saya akan percaya dengan amaran awal HANYA JIKA MAKLUMAT yang disediakan adalah daripada agensi kerajaan
    ☐Yes *Ya* ☐No *Tidak*

25. The government agency should include information of early warning of dengue outbreak as and when they update dengue situations for the public
Agensi kerajaan hendaklah menyertakan maklumat amaran awal wabak denggi apabila mereka mengemaskini maklumat keadaan denggi untuk orang ramai
☐Yes *Ya* ☐No *Tidak* ☐ Not sure *Tidak pasti*

26. We do not need an early warning since weekly dengue situations for my area is available online or social media
Kita tidak memerlukan amaran awal kerana keadaan denggi mingguan untuk kawasan saya boleh didapati 'online' atau daripada media sosial
☐Yes *Ya* ☐No *Tidak* ☐ Not sure *Tidak pasti*

27. It is a waste of time and efforts on dengue control if the predicted risk of dengue outbreak does not come true
Ia adalah satu pembaziran masa dan usaha pada kawalan denggi jika risiko wabak denggi yang diramalkan tidak menjadi kenyataan
☐Yes *Ya* ☐No *Tidak* ☐ Not sure *Tidak pasti*

28. It is pointless for me to take action even with early dengue warning since my neigbours will not
Ia adalah sia-sia bagi saya untuk mengambil tindakan walaupun dengan amaran awal denggi kerana jiran saya tidak akan bertindak
☐Yes *Ya* ☐No *Tidak*

29. The community in my area needs public education about dengue early warning
Masyarakat di kawasan saya perlu diberi pendidikan awam mengenai amaran awal denggi
☐Yes *Ya* ☐No *Tidak* ☐ Not sure *Tidak pasti*

30. I want to know more about how climate can be used to predict a dengue outbreak
Saya ingin mengetahui dengan lebih lanjut mengenai bagaimana maklumat iklim boleh digunakan untuk meramal wabak denggi
☐Yes *Ya* ☐No *Tidak*

31. I would like to receive a periodical update on information of dengue early warning
Saya ingin menerima pengemaskinian berkala tentang maklumat berkenaan amaran awal denggi
☐Yes *Ya* ☐No *Tidak*

32. In what way, would you like to receive an early warning for dengue?
Dengan cara apakah, anda ingin menerima amaran awal denggi?

☐ Mobile APP *Aplikasi mudah alih* ☐ SMS *SMS* ☐ Radio *Radio* ☐ Television *Televisyen*
☐ Facebook *Facebook* ☐ Twitter *Twitter* ☐ Instagram *Instagram*
☐ Other media *Lain-lain media* ____________________________

C) RESPOND TOWARDS AN EARLY WARNING ***MAKLUMBALAS TERHADAP AMARAN AWAL***

33. I check current dengue situations or hotspots around my area regularly
Saya kerap memeriksa situasi denggi semasa atau titik panas di sekitar kawasan saya
☐Yes *Ya* ☐No *Tidak*

34. I do not know what to do if someone informs me that it is very likely to have a dengue outbreak in the near future
Saya tidak tahu apa yang perlu dilakukan jika seseorang memberitahu saya bahawa ia adalah sangat mungkin untuk wabak denggi berlaku dalam masa terdekat
☐Yes *Ya* ☐No *Tidak* ☐ Not sure *Tidak pasti*

35. I will take extra action to prevent dengue infection if I know the risk of dengue is increasing in my area
Saya akan mengambil tindakan tambahan untuk mencegah jangkitan denggi jika saya tahu risiko denggi semakin meningkat di kawasan saya
☐Yes *Ya* ☐No *Tidak* ☐ Not sure *Tidak pasti*

36. After I receive an early warning of dengue outbreak from the government agency, I will:
Selepas saya menerima amaran awal wabak denggi daripada agensi kerajaan, saya akan:

a) Increase source reduction activities
Meningkatkan aktiviti pengurangan sumber
☐Yes *Ya* ☐No *Tidak* ☐ Not sure *Tidak pasti*

b) Avoid outdoor activities at dawn or dusk
Mengelakkan aktiviti luar pada waktu awal pagi atau senja
☐Yes *Ya* ☐No *Tidak* ☐Not sure *Tidak pasti*

c) Share information with others
Berkongsi maklumat dengan orang lain
☐Yes *Ya* ☐No *Tidak* ☐Not sure *Tidak pasti*

d) Request chemical fogging
Membuat permintaan untuk 'chemical fogging'
☐Yes *Ya* ☐No *Tidak* ☐Not sure *Tidak pasti*

e) Call local authorities
Menelefon pihak berkuasa tempatan
☐Yes *Ya* ☐No *Tidak* ☐Not sure *Tidak pasti*

f) Use mosquito net
Menggunakan kelambu
☐Yes *Ya* ☐No *Tidak* ☐Not sure *Tidak pasti*

37. I need to know how severe the predicted dengue outbreak will be in order to decide whether preventive measures are required
Saya perlu tahu bagaimana teruk keadaan wabak denggi yang diramalkan akan berlaku, untuk membuat keputusan sama ada langkah-langkah pencegahan diperlukan atau tidak.
☐Yes *Ya* ☐No *Tidak* ☐ Not sure *Tidak pasti*

38. I will stop action to prevent dengue infection if I know the risk of dengue in my area is low
Saya akan berhenti mengambil tindakan untuk mencegah jangkitan denggi jika saya tahu risiko denggi di kawasan saya adalah rendah
☐Yes *Ya* ☐No *Tidak*

39. The government agency will conduct mosquito control program after they receive an early warning of dengue, so individual household does not need to do anything
Agensi kerajaan akan menjalankan program kawalan nyamuk selepas mereka menerima amaran awal denggi, jadi setiap isi rumah tidak perlu berbuat apa-apa
☐Yes *Ya* ☐No *Tidak* ☐Not sure *Tidak pasti*

40. Removal of mosquitoes breeding sites at my premises will reduce the chance of dengue infections among my family members
Pembuangan tempat pembiakan nyamuk di premis saya akan mengurangkan peluang jangkitan denggi di kalangan ahli keluarga saya
☐Yes *Ya* ☐No *Tidak* ☐Not sure *Tidak pasti*

41. The local authority has already provided sufficient effort on dengue control in my area
Pihak berkuasa tempatan telah memberi usaha yang mencukupi bagi kawalan denggi di kawasan saya
☐Yes *Ya* ☐No *Tidak* ☐Not sure *Tidak pasti*

42. Chemical fogging by the local authority is good enough for us to prevent from dengue infection
Aktiviti 'chemical fogging' oleh pihak berkuasa tempatan adalah sudah cukup baik untuk kami bagi mencegah jangkitan denggi
☐Yes *Ya* ☐No *Tidak* ☐Not sure *Tidak pasti*

43. It is not my responsibility to remove mosquito breeding sites in my residence
Ia bukan tanggungjawab saya untuk menghapuskan tempat pembiakan nyamuk di kediaman saya
☐Yes *Ya* ☐No *Tidak*

44. It is the responsibility of my family member to remove mosquito breeding sites in my residence
Adalah menjadi tanggungjawab ahli keluarga saya untuk menghapuskan tempat pembiakan nyamuk di kediaman saya
☐Yes *Ya* ☐No *Tidak*

45. It is necessary to continue the removal of mosquito breeding at home even during the period when there's no dengue outbreak
Ia adalah perlu untuk meneruskan penghapusan tempat pembiakan nyamuk di rumah walaupun dalam tempoh yang tiada wabak denggi
☐Yes *Ya* ☐No *Tidak* ☐Not sure *Tidak pasti*

46. I can help to reduce dengue cases in my area by removing mosquito breeding sites at home
Saya boleh membantu untuk mengurangkan kes denggi di kawasan saya dengan menghapuskan tempat pembiakan nyamuk di rumah
☐Yes *Ya* ☐No *Tidak* ☐Not sure *Tidak pasti*

47. Dengue outbreak in my community can be controlled if every household is committed to remove mosquito breeding sites
Wabak denggi dalam masyarakat saya boleh dikawal jika setiap isi rumah komited untuk menghapuskan tempat pembiakan nyamuk
☐Yes *Ya* ☐No *Tidak* ☐Not sure *Tidak pasti*

48. I will take part in a public activity for dengue control or removal of mosquitoes breeding sites
Saya akan mengambil bahagian dalam aktiviti awam untuk kawalan denggi atau penghapusan tempat pembiakan nyamuk
☐Yes *Ya* ☐No *Tidak* ☐Not sure *Tidak pasti*

49. In your opinion, who should be responsible for preventing the spread of dengue disease? (You may tick several options)
Pada pendapat anda, siapa yang perlu bertanggungjawab dalam mencegah penyebaran penyakit denggi? (Anda boleh menanda lebih dari satu pilihan)
    ☐Health authority *Pihak berkuasa kesihatan*
    ☐ Local council *Majlis perbandaran*
    ☐Community leaders *Ketua komuniti*
    ☐Every household *Setiap isi rumah tempatan*
    ☐I don´t think control actions are needed *Saya tidak fikir tindakan kawalan adalah perlu*

50. In your opinion, what is the most effective method to reduce dengue infections in your area?
Pada pendapat anda, apakah cara yang paling berkesan untuk mengurangkan jangkitan denggi di kawasan anda?
    ☐Search & destroy mosquito breeding sites
    Mencari dan memusnahkan tempat pembiakan nyamuk
    ☐Prevent from mosquito bites *Mengelakkan gigitan nyamuk*
    ☐Chemical fogging *'Chemical fogging'*
    ☐Don't know *Tidak tahu*

This is the end of survey. Thank you for helping.
Soalan kajiselidik tamat. Terima kasih kerana membantu.
