# Supplementary material for: Public perception and attitude towards dengue prevention activity and response to dengue early warning in Malaysia
Source: PLoS One. 2019 Feb 28;14(2):e0212497. doi: 10.1371/journal.pone.0212497 (PMC6394956; doi:10.1371/journal.pone.0212497)
Supplement: S1 Table — (DOCX) [file pone.0212497.s002.docx]

**Public perception and attitude towards dengue prevention activity and response to dengue early warning in Malaysia**

Rafdzah Zaki^1*^, Siti Norsyuhada Roffeei^1^, Yien Ling Hii^3^, Abqariyah Yahya^1^, Mahesh Appannan^1^, Mas Ayu Said^1^, Ng Chiu Wan^1^, Nasrin Aghamohammadi^2^, Noran Naqiah Hairi^1^, Awang Bulgiba^1^, Mikkel Quam^3^, Joacim Rocklov^3^

**Appendix/Supplementary Material (PONE-D-18-14908)**

**S2 Table. Association of socio-demographic factors with Q18 and Q34.**

| Socio-demographic factors | Q18. Do you think an early warning is a useful tool for community to take preventive actions to avoid possible infection within sufficient time? (N, %) | | | Q34. I do not know what to do if someone informs me that it is very likely to have a dengue outbreak in the near future. (N, %) | | |
| --- | --- | --- | --- | --- | --- | --- |
|  |  |  |  |  |  |  |
|  | **Yes** | **No** | ***p*** | **Yes** | **No** | ***p*** |
| 1. Gender |  |  |  |  |  |  |
| Male | 238 (80.4) | 58 (19.6) | 0.918 | 95 (32.5) | 197 (67.5) | 0.073 |
| Female | 439 (80.7) | 105 (19.3) |  | 145 (26.7) | 399 (73.3) |  |
| 2. Age |  |  |  |  |  |  |
| ≤40 years | 594 (79.7) | 151 (20.3) | 0.061 | 218 (29.3) | 525 (70.7) | 0.056 |
| >40 years | 64 (88.9) | 8 (11.1) |  | 13 (18.6) | 57 (81.4) |  |
| 3. Nationality |  |  |  |  |  |  |
| Malaysian | 661 (80.6) | 159 (19.4) | 0.831 | 231 (28.3) | 585 (71.7) | 0.072 |
| Non-Malaysian | 7 (77.8) | 2 (22.2) |  | 5 (55.6) | 4 (44.4) |  |
| 4. Race |  |  |  |  |  |  |
| Malay | 501 (80.8) | 119 (19.2) | 0.234 | 172 (27.9) | 444 (72.1) | 0.596 |
| Chinese | 57 (89.1) | 7 (10.9) |  | 21 (32.8) | 43 (67.2) |  |
| Indian | 73 (78.5) | 20 (21.5) |  | 23 (24.5) | 71 (75.5) |  |
| Others | 32 (74.4) | 11 (25.6) |  | 14 (33.3) | 28 (66.7) |  |
| 5. Do you live in Petaling district? | | |  |  |  |  |
| Yes | 296 (82.9) | 61 (17.1) | 0.133 | 121 (33.9) | 236 (66.1) | 0.004 |
| No | 378 (78.8) | 102 (21.3) |  | 118 (24.8) | 358 (75.2) |  |
| 5(a). Do you study/work in Petaling district? | | | |  |  |  |
| Yes | 342 (81.2) | 79 (18.8) | 0.070 | 131 (31.1) | 290 (68.9) | 0.376 |
| No | 156 (75.0) | 52 (25.0) |  | 57 (27.7) | 149 (72.3) |  |
| 5(b). Period living in Petaling district? | | | |  |  |  |
| ≤3 years | 194 (76.7) | 59 (23.3) | 0.085 | 80 (31.7) | 172 (68.3) | 0.613 |
| >3 years | 199 (82.9) | 41 (17.1) |  | 81 (33.9) | 158 (66.1) |  |
| 5(c). Do you own current residence? | | |  |  |  |  |
| Yes | 159 (82.0) | 35 (18.0) | 0.306 | 56 (29.3) | 135 (70.7) | 0.623 |
| No | 255 (78.2) | 71 (21.8) |  | 102 (31.4) | 223 (68.6) |  |
| 6. What type of house you currently reside? | | | |  |  |  |
| Individual house or bungalow | 56 (70.9) | 23 (29.1) | 0.200 | 20 (25.3) | 59 (74.7) | 0.656 |
| Twin/semi-detached house | 50 (86.2) | 8 (13.8) |  | 15 (26.8) | 41 (73.2) |  |
| Terrace house | 239 (83.0) | 49 (17.0) |  | 79 (27.7) | 206 (72.3) |  |
| Flat | 88 (79.3) | 23 (20.7) |  | 40 (35.7) | 72 (64.3) |  |
| Apartment/condominium | 145 (79.7) | 37 (20.3) |  | 48 (26.2) | 135 (73.8) |  |
| Shophouse/long house/others | 89 (80.2) | 22 (19.8) |  | 35 (31.8) | 75 (68.2) |  |
| 7. Education |  |  |  |  |  |  |
| <Tertiary education | 120 (75.9) | 38 (24.1) | 0.097 | 47 (30.3) | 108 (69.7) | 0.657 |
| Tertiary education | 551 (81.8) | 123 (18.2) |  | 192 (28.5) | 481 (71.5) |  |
| 8. Marital status | |  |  |  |  |  |
| Single | 460 (78.6) | 125 (21.4) | 0.039 | 183 (31.4) | 399 (68.6) | 0.072 |
| Married | 204 (86.1) | 33 (13.9) |  | 53 (22.5) | 183 (77.5) |  |
| Divorced | 6 (66.7) | 3 (33.3) |  | 2 (22.2) | 7 (77.8) |  |
| Widow/widower | 5 (100.0) | 0 (0.0) |  | 1 (20.0) | 4 (80.0) |  |
| 9. Occupation |  |  |  |  |  |  |
| Student | 340 (77.3) | 100 (22.7) | 0.005 | 133 (30.4) | 305 (69.6) | 0.528 |
| Self-employed | 63 (78.8) | 17 (21.3) |  | 17 (21.8) | 61 (78.2) |  |
| Government workers | 97 (85.1) | 17 (14.9) |  | 31 (26.7) | 85 (73.3) |  |
| Private workers | 146 (89.0) | 18 (11.0) |  | 47 (29.0) | 115 (71.0) |  |
| Unemployed | 19 (67.9) | 9 (32.1) |  | 7 (25.0) | 21 (75.0) |  |
| Other | 6 (100.0) | 0 (0.0) |  | 3 (50.0) | 3 (50.0) |  |
| 10. Have you had dengue fever before? | | | |  |  |  |
| Yes | 137 (85.1) | 24 (14.9) | 0.125 | 39 (24.2) | 122 (75.8) | 0.154 |
| No | 537 (79.8) | 136 (20.2) |  | 200 (29.9) | 469 (70.1) |  |
| 11. Do you know any persons who have been infected with dengue? | | | | | |  |
| Yes | 536 (82.8) | 111 (17.2) | 0.003 | 179 (27.8) | 465 (72.2) | 0.192 |
| No | 132 (72.9) | 49 (27.1) |  | 59 (32.8) | 121 (67.2) |  |
| 12. Number of people in your household? | | | |  |  |  |
| ≤5 people | 457 (83.1) | 93 (16.9) | 0.073 | 157 (28.6) | 391 (71.4) | 0.693 |
| >5 people | 204 (77.9) | 58 (22.1) |  | 78 (30.0) | 182 (70.0) |  |
| 13. Average monthly income? | | |  |  |  |  |
| ≤MYR3000 | 257 (80.1) | 64 (19.9) | 0.004 | 106 (33.0) | 215 (67.0) | 0.059 |
| > MYR3000 | 122 (91.0) | 12 (9.0) |  | 32 (24.1) | 101 (75.9) |  |
| 14. Average household monthly income? | | | |  |  |  |
| ≤ MYR6000 | 319 (82.6) | 67 (17.4) | 0.754 | 114 (29.4) | 274 (70.6) | 0.400 |
| > MYR6000 | 144 (83.7) | 28 (16.3) |  | 56 (32.9) | 114 (67.1) |  |

*MYR = Malaysian Ringgit
