# Supplementary material for: Public perception and attitude towards dengue prevention activity and response to dengue early warning in Malaysia
Source: PLoS One. 2019 Feb 28;14(2):e0212497. doi: 10.1371/journal.pone.0212497 (PMC6394956; doi:10.1371/journal.pone.0212497)
Supplement: S2 Table — (DOCX) [file pone.0212497.s003.docx]

**Public perception and attitude towards dengue prevention activity and response to dengue early warning in Malaysia**

Rafdzah Zaki^1*^, Siti Norsyuhada Roffeei^1^, Yien Ling Hii^3^, Abqariyah Yahya^1^, Mahesh Appannan^1^, Mas Ayu Said^1^, Ng Chiu Wan^1^, Nasrin Aghamohammadi^2^, Noran Naqiah Hairi^1^, Awang Bulgiba^1^, Mikkel Quam^3^, Joacim Rocklov^3^

**Appendix/Supplementary Material (PONE-D-18-14908)**

**S3 Table. Association of perception (Section A) with Q18 and Q34.**

| Perception (Section A) | Q18. Do you think an early warning is a useful tool for community to take preventive actions to avoid possible infection within sufficient time?  (N, %) | | | Q34. I do not know what to do if someone informs me that it is very likely to have a dengue outbreak in the near future.  (N, %) | | |
| --- | --- | --- | --- | --- | --- | --- |
|  |  |  |  |  |  |  |
|  | **Yes** | **No** | ***p*** | **Yes** | **No** | ***p*** |
| 1. Do you know what dengue fever is? | | |  |  |  |  |
| Yes | 663 (81.7) | 149 (18.3) | <0.001 | 228 (28.3) | 579 (71.7) | 0.152 |
| No | 12 (50.0) | 12 (50.0) |  | 10 (41.7) | 14 (58.3) |  |
| 2. Do you think dengue fever can cause mortality? | | | |  |  |  |
| Yes | 657 (81.6) | 148 (18.4) | 0.004 | 225 (28.1) | 575 (71.9) | 0.103 |
| No | 5 (45.5) | 6 (54.5) |  | 4 (36.4) | 7 (63.6) |  |
| Don't know | 16 (69.6) | 7 (30.4) |  | 11 (47.8) | 12 (52.2) |  |
| 3. Do you think you and your family members could be infected with dengue fever? | | | | | | |
| Yes | 546 (82.9) | 113 (17.1) | 0.010 | 192 (29.3) | 463 (70.7) | 0.375 |
| No | 43 (71.7) | 17 (28.3) |  | 21 (34.4) | 40 (65.6) |  |
| Don't know | 82 (73.2) | 30 (26.8) |  | 27 (24.5) | 83 (75.5) |  |
| 4. In your opinion, what is your risk of being infected with dengue fever? | | | | | |  |
| Low | 157 (83.5) | 31 (16.5) | 0.438 | 47 (25.3) | 139 (74.7) | 0.491 |
| Medium | 368 (80.7) | 88 (19.3) |  | 134 (29.6) | 318 (70.4) |  |
| High | 148 (78.3) | 41 (21.7) |  | 57 (30.0) | 133 (70.0) |  |
| 5. Do you think you have sufficient knowledge of the ways to prevent yourself from dengue infection? | | | | | | |
| Yes | 452 (84.3) | 84 (15.7) | <0.001 | 145 (27.2) | 389 (72.8) | 0.021 |
| No | 161 (77.4) | 47 (22.6) |  | 74 (35.9) | 132 (64.1) |  |
| Don't know | 63 (67.0) | 31 (33.0) |  | 21 (22.3) | 73 (77.7) |  |
| 6. Do you think the dengue situation is serious in the area you live in? | | | | | |  |
| Yes | 326 (85.6) | 55 (14.4) | 0.001 | 122 (32.1) | 258 (67.9) | 0.076 |
| No | 233 (79.3) | 61 (20.7) |  | 71 (24.1) | 223 (75.9) |  |
| Don't know | 118 (72.0) | 46 (28.0) |  | 47 (29.2) | 114 (70.8) |  |
| 7. Do you think it is possible to be infected with dengue many times? | | | | | |  |
| Yes | 357 (86.0) | 58 (14.0) | <0.001 | 119 (28.8) | 294 (71.2) | 0.929 |
| No | 136 (73.5) | 49 (26.5) |  | 52 (28.0) | 134 (72.0) |  |
| Don't know | 184 (77.0) | 55 (23.0) |  | 70 (29.7) | 166 (70.3) |  |
| 8. How concerned would you be if it was the second time or more for your parents/children to be infected with dengue? | | | | | | |
| Very concerned | 566 (82.7) | 118 (17.3) | <0.001 | 185 (27.2) | 496 (72.8) | 0.079 |
| Concerned | 81 (72.3) | 31 (27.7) |  | 43 (38.7) | 68 (61.3) |  |
| Slightly concerned | 25 (80.6) | 6 (19.4) |  | 10 (32.3) | 21 (67.7) |  |
| Not concerned | 2 (20.0) | 8 (80.0) |  | 2 (20.0) | 8 (80.0) |  |
| 9. Which methods can be used to protect yourself and your family members from dengue infection? | | | | | | |
| 9.1) Nothing |  |  |  |  |  |  |
| Yes | 5 (62.5) | 3 (37.5) | 0.193 | 2 (25.0) | 6 (75.0) | 0.810 |
| No | 672 (80.8) | 160 (19.2) |  | 239 (28.9) | 589 (71.1) |  |
| 9.2) Don't know | |  |  |  |  |  |
| Yes | 17 (51.5) | 16 (48.5) | <0.001 | 17 (54.8) | 14 (45.2) | 0.001 |
| No | 660 (81.8) | 147 (18.2) |  | 224 (27.8) | 581 (72.2) |  |
| 9.3) Mosquito repellent | |  |  |  |  |  |
| Yes | 477 (83.2) | 96 (16.8) | 0.004 | 149 (26.0) | 423 (74.0) | 0.009 |
| No | 200 (74.9) | 67 (25.1) |  | 92 (34.8) | 172 (65.2) |  |
| 9.4) Insecticide | |  |  |  |  |  |
| Yes | 295 (84.3) | 55 (15.7) | 0.022 | 87 (25.0) | 261 (75.0) | 0.039 |
| No | 382 (78.0) | 108 (22.0) |  | 154 (31.6) | 334 (68.4) |  |
| 9.5) Bed nets |  |  |  |  |  |  |
| Yes | 336 (85.7) | 56 (14.3) | <0.001 | 83 (21.3) | 307 (78.7) | <0.001 |
| No | 341 (76.1) | 107 (23.9) |  | 158 (35.4) | 288 (64.6) |  |
| 9.6) Remove mosquito breeding sites | | |  |  |  |  |
| Yes | 585 (83.2) | 118 (16.8) | <0.001 | 173 (24.7) | 528 (75.3) | <0.001 |
| No | 92 (67.2) | 45 (32.8) |  | 68 (50.4) | 67 (49.6) |  |
| 9.7) Others |  |  |  |  |  |  |
| Yes | 90 (77.6) | 26 (22.4) | 0.377 | 26 (22.4) | 90 (77.6) | 0.100 |
| No | 587 (81.1) | 137 (18.9) |  | 215 (29.9) | 505 (70.1) |  |
| 10. Do you think the global climate is changing? | | | |  |  |  |
| Yes | 586 (83.5) | 116 (16.5) | <0.001 | 193 (27.5) | 508 (72.5) | 0.003 |
| No | 28 (65.1) | 15 (34.9) |  | 22 (52.4) | 20 (47.6) |  |
| Don't know | 62 (66.0) | 32 (34.0) |  | 26 (28.0) | 67 (72.0) |  |
| 11. Do you think the climate change does not influence Malaysia climate? | | | | | |  |
| Yes | 150 (81.1) | 35 (18.9) | <0.001 | 74 (39.8) | 112 (60.2) | 0.001 |
| No | 424 (84.6) | 77 (15.4) |  | 128 (25.6) | 372 (74.4) |  |
| Don't know | 101 (66.4) | 51 (33.6) |  | 39 (26.0) | 111 (74.0) |  |
| 12. Do you think the climate change affects human health? | | | | |  |  |
| Yes | 633 (83.4) | 126 (16.6) | <0.001 | 208 (27.5) | 548 (72.5) | 0.041 |
| No | 15 (60.0) | 10 (40.0) |  | 12 (48.0) | 13 (52.0) |  |
| Don't know | 28 (51.9) | 26 (48.1) |  | 19 (35.8) | 34 (64.2) |  |
| 13. Do you think the global warming could increase the risk of dengue outbreaks? | | | | | | |
| Yes | 487 (84.5) | 89 (15.5) | <0.001 | 174 (30.4) | 399 (69.6) | 0.302 |
| No | 53 (72.6) | 20 (27.4) |  | 17 (23.6) | 55 (76.4) |  |
| Don't know | 135 (71.8) | 53 (28.2) |  | 49 (25.9) | 140 (74.1) |  |
| 14. Do you think the climatic factors may affect the life cycle of mosquitoes but not dengue cases? | | | | | | |
| Yes | 323 (80.5) | 78 (19.5) | 0.001 | 136 (34.0) | 264 (66.0) | 0.007 |
| No | 174 (88.3) | 23 (11.7) |  | 49 (24.9) | 148 (75.1) |  |
| Don't know | 177 (74.4) | 61 (25.6) |  | 55 (23.5) | 179 (76.5) |  |
| 15. Do you think the number of dengue cases increases after rainy days? | | | | | |  |
| Yes | 535 (83.9) | 103 (16.1) | <0.001 | 187 (29.5) | 447 (70.5) | 0.307 |
| No | 48 (75.0) | 16 (25.0) |  | 21 (32.8) | 43 (67.2) |  |
| Don't know | 92 (67.6) | 44 (32.4) |  | 32 (23.7) | 103 (76.3) |  |
| 16. Do you think the increasing temperature elevates the number of dengue cases in your area? | | | | | | |
| Yes | 412 (84.9) | 73 (15.1) | 0.001 | 161 (33.5) | 319 (66.5) | 0.001 |
| No | 82 (75.2) | 27 (24.8) |  | 29 (26.4) | 81 (73.6) |  |
| Don't know | 182 (74.3) | 63 (25.7) |  | 50 (20.5) | 194 (79.5) |  |
| 17. Do you think the information about previous temperature and rainfall can be used to predict dengue outbreak in future? | | | | | | |
| Yes | 443 (87.9) | 61 (12.1) | <0.001 | 145 (28.9) | 356 (71.1) | 0.015 |
| No | 68 (78.2) | 19 (21.8) |  | 35 (40.7) | 51 (59.3) |  |
| Don't know | 166 (66.7) | 83 (33.3) |  | 60 (24.3) | 187 (75.7) |  |
| 18. Do you think an early warning is a useful tool for community to take preventive actions to avoid possible infection within sufficient time? | | | | | | |
| Yes | . | . | . | 196 (29.2) | 475 (70.8) | 0.008 |
| No | . | . |  | 25 (40.3) | 37 (59.7) |  |
| Don't know | . | . |  | 18 (18.0) | 82 (82.0) |  |
